# Modafinil Increases the Latency of Response in the Hayling Sentence Completion Test in Healthy Volunteers: A Randomised Controlled Trial

**DOI:** 10.1371/journal.pone.0110639

**Published:** 2014-11-12

**Authors:** Ahmed Dahir Mohamed, Chris Roberts Lewis

**Affiliations:** 1 Department of Psychiatry, School of Clinical Medicine, University of Cambridge, Cambridge, United Kingdom; 2 The School of Psychology, Cognitive and Sensory Systems Group, Faculty of Science, University of Nottingham Malaysia Campus, Selangor, Malaysia; 3 Clare Hall College, Cambridge, United Kingdom; 4 Department of Psychology, Towson University, 8000 York Road, Towson, Maryland, 21252-0001, United States of America; Monash University, Australia

## Abstract

**Background:**

Modafinil is a medication licensed for the treatment of narcolepsy. However, it has been reported that healthy individuals without wakefulness disorders are using modafinil off-label to enhance cognitive functioning. Although some studies have reported that modafinil improves cognitive task performance in healthy volunteers, numerous other studies have failed to detect cognitive enhancing effects of modafinil on several well-established neuropsychological tasks. Interestingly, several clinical and preclinical studies have found that improved cognitive task performance by modafinil is accompanied by slower response times. This observation raises the question as to whether this slowing of response time in healthy volunteers is a necessary and sufficient condition for cognitive enhancement with modafinil. The aim of the current experiment was to explore this question by investigating the effects of modafinil on the Hayling Sentence Completion Test (HSCT).

**Methodology:**

Sixty-four healthy volunteers received either a single dose (200 mg) of modafinil (n = 32) or placebo (n = 32) in a randomized, double-blind, placebo-controlled, parallel group study in which the principal outcome measures were response latencies on the response initiation and response inhibition sections of the HSCT.

**Principal Findings:**

Participants dosed with modafinil had significantly longer mean response latencies on the HSCT for both the response initiation and response inhibition compared to participants dosed with placebo. However, participants in both groups made a similar number of errors on each of these measures, indicating that modafinil did not enhance the accuracy of performance of the task relative to placebo.

**Conclusions:**

This study demonstrated that administration of single 200 mg doses of modafinil to healthy individuals increased the latency of responses in the performance of the HSCT, a task that is highly sensitive to prefrontal executive function, without enhancing accuracy of performance. This finding may provide important clues to defining the limitations of modafinil as a putative cognitive enhancer.

**Trial Registration:**

ClinicalTrials.gov NCT02051153

## Introduction

Modafinil is a wakefulness-promoting medication licenced for the treatment of narcolepsy, as well as several other disorders of wakefulness [1]. Experimental studies have reported that modafinil improves the performance of working memory and planning tasks in healthy volunteers, and in patients with neuropsychiatric disorders [2]. More specifically, several studies have reported improvements in some, but not all, neuropsychological tasks, which assess cognitive functions such as memory, visuo-spatial skills, planning and attention [3]–[5]. These experimental findings have led researchers [3], [4], [6] and media reports [7], [8] to suggest that modafinil may be acting as a neurocognitive enhancer in healthy individuals.

Currently, the global market share of modafinil is more than US$700 million per year [9] and it has been estimated that around 90% of modafinil is predominantly used off-label by healthy, non-sleep deprived individuals who are aiming not to just get ‘high’, but to increase attention and wakefulness [10], [11]. Several research reports indicate that a significant number of healthy individuals without wakefulness disorders have used modafinil off-label with the intention of improving their cognitive functioning, for example, to boost academic [10]–[12] or job performance [13]. Consistent with these reports, it has been claimed that increasing numbers of healthy physicians on-call, students, and academic professionals are using modafinil with the aim of enhancing their cognitive abilities [13], [14].

Nevertheless, double-blind, placebo-controlled experimental trials in healthy volunteers have failed to find cognitive-enhancing effects of modafinil on several well-established neuropsychological tasks [15]–[17]. For example, Müller et al. [3] failed to find effects of 200 mg of modafinil on simple digit maintenance, letter cancellation and trail making, while Turner et al. [18] reported that 100 mg and 200 mg of modafinil did not have any significant effects on spatial memory span, spatial working memory, rapid visual processing or attentional set-shifting task performance. Additionally, no beneficial effects of modafinil were observed on the paired associates learning task, which assesses memory, and the ‘one-touch’ Tower of London spatial planning task. Furthermore, Turner et al. [18] also reported no effects of modafinil on the Cambridge Gambling task.

Other subsequent studies showed that higher doses of modafinil also seem to be unbeneficial for cognitive improvements in healthy, non-sleep deprived individuals. For instance, Winder-Rhodes et al. [5] showed that 300 mg of modafinil failed to have an effect on a planning task. Similarly, Baranski at al. [10] also used high doses of modafinil (4 mg/kg or 300 mg) with healthy volunteers and found no cognitive enhancing effects of modafinil on addition, line discrimination and subjective confidence judgment during the performance on cognitive tasks. Likewise, Wesensten et al. [19] reported lack of effects of 400 mg of modafinil over and above 600 mg of caffeine on the Stroop, verbal fluency, simple reaction time, and the Wisconsin's Card Sorting task. More importantly, several studies failed to replicate Turner et al.'s [18] positive findings in the same neuropsychological tasks with healthy non-sleep deprived individuals [15]–[17]. Winder-Rhodes et al. [5] argued that the influence of modafinil may be subtle, restricted to challenging tasks and limited in healthy non-sleep-deprived participants.

Consistent with these negative findings, a series of studies with healthy volunteers by Randall et al. [15]–[17] failed to find any effect of 100 or 200 mg of modafinil on spatial working memory, logic memory, the Paced Auditory Serial Addition Test, symbol copy, digit cancellation, verbal fluency, ID/ED-an attentional set-shifting task, trail A/B task, verbal fluency, clock drawing, visual delayed matching to sample, spatial planning, digit span, sustained attention, logical memory, Stroop and verbal fluency tasks.

Turner et al., [20] reported that modafinil improved cognition and response inhibition in adults diagnosed with Attention-Deficit Hyperactivity Disorder (ADHD), a condition often characterized by impulsivity, inattention, and hyperactivity. However, Turner et al. [20] found that the observed sustained attention and accuracy by modafinil was due to the drug slowing response latency. This is consistent with earlier evidence from healthy non-sleep deprived individuals suggesting that, relative to placebo, modafinil increases performance accuracy in conjunction with a slowing of response latency on several tasks [18].

Pre-clinically, there is also evidence that treatment of mice with modafinil produces a delay-dependent enhancement in spatial working memory [21], a cognitive function that depends critically upon alertness and attention. However, in a subsequent study of the role of modafinil as a potential enhancer of attentional processes in rat, Waters et al., [22] reported that modafinil failed to significantly enhance five-choice serial reaction time test performance under standard conditions. Similarly, modafinil was unable to reverse the deficits in accuracy and/or increased omission errors induced by either parametric or pharmacological manipulations. Indeed, at higher doses, it was reported that modafinil caused an increase in premature responding or impulsivity under certain test conditions. These investigators concluded that they had found no evidence to support a modafinil-induced improvement in response control; rather, under conditions of increased attentional load, modafinil appeared to facilitate impulsive responding [22].

One explanation for the lack of cognitive enhancing effects of modafinil in these studies might be that the drug could have highly cognitive domain-specific effects, and thereby affect neuropsychological test performance only to the extent that such domains are critically involved in the performance on that test. Alternatively, it is possible that modafinil does not improve cognition in a meaningful way in most healthy individuals. Conceivably, positive results in some studies could be due to the presence of sufficient numbers of individuals with a relatively low level of alertness, such that the known wakefulness-enhancing effect of the drug improves performance on certain tasks that are relatively sensitive to modest decrements in alertness. The foregoing negative studies, in addition to other evidence supporting the notion that modafinil does not always improve or enhance several neuropsychological task measures [2], [23], raise the possibility that modafinil may be mechanistically unable to enhance certain task-specific, sophisticated problem-solving abilities in healthy individuals [24].

These conflicting results challenge the view that modafinil is a “broad spectrum” cognitive enhancer, particularly in humans who exhibit presumably neurotypical levels of cognitive function and alertness/arousal. Moreover, the mechanisms by which modafinil exerts its known therapeutic effects in patients with narcolepsy are still unclear [2], [25]. Furthermore, despite the high level of reported off-label use of modafinil as a cognitive enhancer [26], the mechanisms by which modafinil modulates cognitive and affective processes in healthy individuals are currently unknown and need to be thoroughly investigated [27]–[30]. Crucially, and more specifically, it is still unclear how modafinil affects cognitive domains that involve response initiation and inhibition in healthy individuals.

In view of the foregoing evidence that modafinil increases performance accuracy in conjunction with a slowing of response latency on several tasks, an outstanding question regarding the effects of modafinil on cognitive responses is whether the drug generally increases the latency of response to all cognitive tasks or whether it increases the latency of response only for tasks that measure impulsivity and require cognitive and behavioural control. If modafinil is acting selectively as a cognitive enhancer, the drug would be expected to reduce impulsivity in tasks that require preparedness and cognitively controlled responses while not slowing the responses during tasks that require both planning and quick decision making. However, if modafinil is acting non-selectively to slow the rate of responding, regardless of the task requirements, then the apparent cognitive performance-enhancing effects of the drug for certain tasks might be more correctly understood as an indirect effect rather than a direct cognition-enhancing mode of action.

The aim of the current experiment was to address this question by investigating the effects of modafinil on the HSCT, a robust neuropsychological measure of response initiation and response inhibition [31] that is highly sensitive to frontal lobe dysfunction [32], [33]. Previous neuropsychological research has consistently shown that patients with neuropsychiatric disorders and those with brain damage perform significantly worse on the HSCT than neurologically healthy individuals [31], [34], as do patients with narcolepsy [35]. The HSCT requires quick and accurate responses and activates brain areas that are dependent on language retrieval, semantic activation and selection in semantic search [36]. For example, studies using positron emission tomography (PET) [37] and functional magnetic resonance imaging (fMRI) [38] that examined the cortical areas involved during the performance of the HSCT have shown that response initiation processes are associated with increases of activity in the left inferior frontal gyrus (BA 10/45/47), whereas response inhibition processes increased the left prefrontal areas, including the middle (BA 9 and BA 10) and inferior (BA 45) frontal areas. The left frontal areas (BA 45) are important for semantic response selection when there are numerous alternatives available [39]. Hence, we used the HSCT to investigate the effects of modafinil on response inhibition and response initiation in healthy volunteers. Based on the above reviewed studies, we hypothesized that modafinil will increase the latency of response to the HSCT. Furthermore, we hypothesized that modafinil will not improve accuracy of the HSCT.

## Methods

### Ethical approval

The protocol for this trial and supporting CONSORT checklist are available as supporting information; see Checklist S1 and Protocol S1.

The study was approved by the East of England–Cambridge Central Research and Ethics Committee (LRECT No: 10/H0305/39) and the Medicines and Health Care Products Regulatory Agency [40], the national drug licensing agency, London. All clinical investigations were conducted according to the principles expressed in the Declaration of Helsinki. The drug and the placebo tablets, which were identical, were synthesised in the Royal Free Hospital, London.

**Figure 1:**
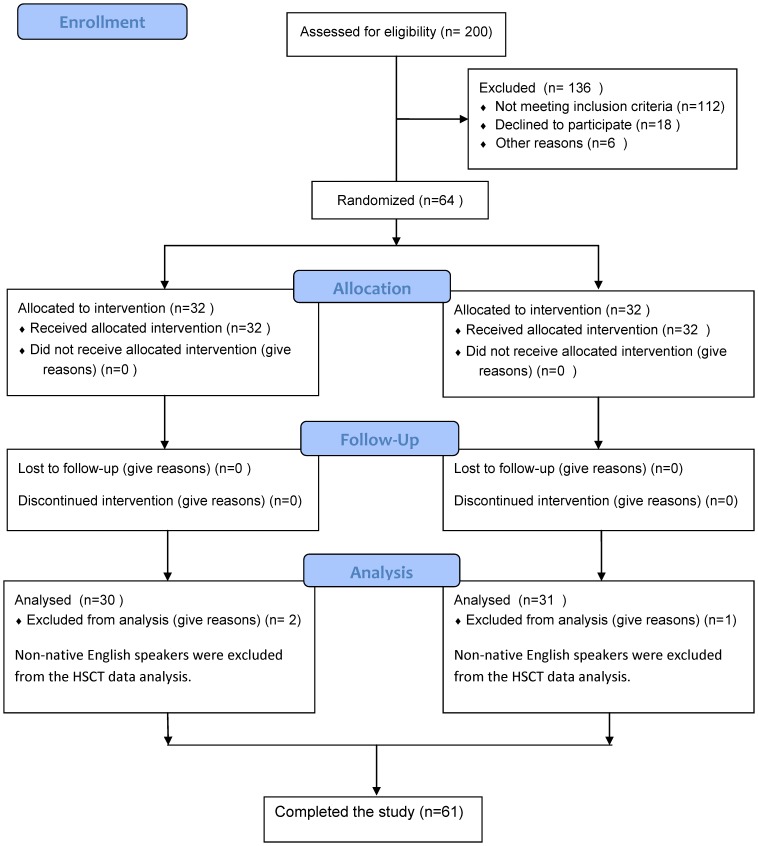
CONSORT Diagram. Flow diagram graphically describes the design of the study: enrolment, intervention, follow-up and data analysis.

As this study was looking at the effects of modafinil on cognitive performance in healthy participants, the ethics committee approved only an acute (200 mg) of modafinil. The study was not considered a clinical trial by the MHRA in the UK because it was deemed as a proof of concept study. Therefore, the study was not initially registered as a clinical trial. The authors confirm that all on-going and related trials for this drug/intervention are now registered. This study was part of a larger clinical and cognitive neuroscience programme consisting of several experiments that were conducted as part of a PhD study. See **Figure 1** for the study group and experimental design.

**Protocol ID**: NCT02051153:

http://clinicaltrials.gov/ct2/show/NCT02051153

### Population study and design

Sixty-four healthy male (n = 31) and female (n = 33) volunteers (Mean age ± SD = 25.34±3.95, range 19–36 years) were identified via the City of Cambridge participant panel, and via local advertisements. A qualified psychologist screened all volunteers for the presence of any of the pre-specified exclusion criteria. Participants were excluded if they had any significant psychiatric history, visual or motor impairment, or the concurrent use of any psychotropic medications or any medication contraindicated with modafinil. In addition, participants with a history of hypertension, cardiac disorders, epilepsy, and drug or alcohol abuse were also excluded. All participants were advised not to consume alcohol or caffeine for 12 hours before the testing sessions. A computer, using separate randomization schedules for each drug (modafinil or placebo), performed the randomization (see Randomization and Masking). All participants were questioned about compliance with alcohol and caffeine restrictions before inclusion into the study. A light breakfast or snack and juice were allowed before, but not during the experimental session. Each participant gave a written informed consent prior to testing and received monetary compensation of £25, plus local transport expenses. After participants were assessed by the psychologist, they then completed baseline physiological measures (blood pressure and pulse rate) and the National Adult Reading Test (NART), which is used to calculate estimated IQ and matches participants' level of verbal IQ [41]. Two consultant neurologists signed off the modafinil medications and the placebo before they were administered and were standing by in case of any adverse effects. Participants were then given a single oral dose (200 mg) of modafinil (n = 32) or placebo (n = 32) with a small glass of water. They were then asked to rest in a quiet room. Two hours post-drug administration, participants completed the HSCT. Participants also completed an hourly visual analogue mood scale at four hourly points. After the completion of the study, participants were debriefed by the researcher and were discharged by a research nurse. Participant recruitment for the study took place between October 2009 and August 2011.

### The Hayling Sentence Completion Test (HSCT)

The HSCT is used because it is a highly sensitive neuropsychological measure of frontal lobe function [31]–[33] that tap into response initiation and suppression of words. These are important cognitive functions that need to be investigated with a putative cognitive enhancing drug. In this experiment, the task was used to measure the effects of modafinil on cognitive flexibility, response inhibition and response initiation in the domain of language retrieval, semantic activation and selection in semantic search. The HSCT has recently been shown to be independent of fluid intelligence performance [42]. Studies from anterior brain damaged patients, and healthy participants, showed that the split-half reliability for the HSCT was 0.92 for time 1, 0.82 for time 2, and 0.72 for time 3 [31]. Similarly, for HSCT errors, section test retest reliability for healthy volunteers was 0.62 for time 1, 0.78 for time 2, and 0.52 for time 3. Overall, test retest reliability for the task was 0.72, indicating high reliability.

In this study, the task consisted of 30 sentences, each missing the last word, which was constructed to strongly constrain what the missing word should be. In the first section (automatic completion section), participants were asked to listen carefully to each sentence, and were asked to provide, as quickly as possible, a word that correctly and sensibly completed the sentence. In the remaining sentences (inhibition completion section), participants were asked to complete the sentences, as quickly as possible, with words unrelated to the meaning of the sentences in every way. Participants' responses and reaction times were recorded. The response times were captured by the experimenter with a stopwatch (model: Acctim Tim901r) with 1/100 second lap and split timing. Both sections were scored separately first, and then together, to yield a total score.

### Visual analogue scales (VAS, Mood)

The visual analogue scale (VAS, Bond and Lader [43]) is a rapid, highly reliable (α = 0.76) measure that assesses transient changes in participants feelings [44]. Its reliability has been compared with the well-validated Hamilton Scale and was found to be 0.79 [45]. The VAS has been used in previous modafinil studies with healthy individuals [18].

Participants completed the VAS before administration of the drug (baseline) and at intervals during the testing session: immediately prior to testing (2 hours post dosing), 1 h into testing (3 hours post dosing) and on completion of testing (discharge). At each time point, participants were asked to rate their feelings in terms of 16 dimensions. The measures used in this study were alert–drowsy, calm–excited, strong–feeble, muzzy–clear headed, well-coordinated–clumsy, lethargic–energetic, contented–discontented, troubled–tranquil, mentally slow–quick witted, tense–relaxed, attentive–dreamy, incompetent–proficient, happy–sad, antagonistic–amicable, interested–bored and withdrawn–gregarious. The dimensions were presented as 100-mm lines, with the two extremes of the feeling (e.g. ‘alert’ and ‘drowsy’) written at each end, and participants marked where they felt they ranked on each line. All factors were analysed and reported.

### The National Adult Reading Test (NART)

This test allows the calculation of pre-morbid IQ estimates and matches participants' level of verbal IQ [46]. NART is amongst one of the most reliable clinical tests for pre-morbid IQ estimates [47], [48]. Its internal consistency is >0.90 [49], [50].

### Cardiovascular measures

Blood pressure and pulse measurements were taken using a Criticare Systems Inc. Comfort Cuff (Model 507NJ) at five time points: immediately upon arrival, before drug administration, immediately prior to testing (2 h post-drug), 1 h into testing (3 h post-drug), and on completion of the study (4 h post-drug). These cardiovascular measures were collected for subject safety, to ensure that no subjects experienced or would be discharged with medically significant changes in blood pressure or heart rate.

### Randomization and Masking

Randomisation was undertaken by a computer using separate schedules for each condition. The experimenter, psychologist and neurologists were blind to drug condition. Condition allocation was known to the nurses in the clinical facility, who physically administered the drug to the participants, as well as a senior psychiatrist and the clinical facility manager who were periodically monitoring, but not involved in, the conduct of the study.

### Statistical significance

Based on use of the statistical power software, G-Power, a sample size of 30 participants per treatment group was calculated to provide a power of 0.95 to detect a large effect size with ANOVA (**η** ***p*** **^2^** = 0.40) and with 0.05 chance of a Type I error (i.e., false positive error of 5%).

### Statistical analysis

All data were analysed using Windows Version 15 of SPSS. For the demographic data (age, years of education, verbal intelligence as measured by the NART), independent sample t-tests were used to determine whether there were any significant differences between the two experimental groups for these characteristics. For gender, a Chi-squared test was used to assess whether the percentage of participants that were in the modafinil or placebo group differed significantly by gender.

The VAS and the cardiovascular measures were separately analysed with a two way mixed-model analysis of variance (ANOVA) using the drug (modafinil vs. placebo) as between-subject variable and the hourly measures as within-subject variable (repeated measures analysis) to investigate any significant differences between the two group means, the hourly mean scores, and any interaction between the hourly mean scores and the treatment intervention. As there were two parallel treatment groups (modafinil and placebo) who were sequentially tested on the Response Initiation and Response Inhibition sections of the HSCT, differences between group mean performance on the HSCT were analysed using a two-way, mixed-model ANOVA with repeated measures for both response latencies and response errors [51], [52]. The partial eta squared values (η*p* ^2^), which provide a measure of effect size for ANOVAs [53], are reported.

## Results

### Demographic, cardiovascular, and mood variables

Following randomization, the two treatment groups were not statistically significantly different in years of education (t(62) = 0.781, p = 0.43), age (t(62) = 1.691, p = 0.096), or verbal intelligence (as measured with the NART; t(62) = 1.532, p = 0.13). Furthermore, the percentage of participants who took modafinil or placebo did not significantly differ by gender (χ2(1, N = 64)  = 0.00, p = 0.99). (**Table 1**).

**Table 1:** Demographic Measures.

Demographics	Modafinil	Placebo	t(df)	P value
	Mean (SD)	Mean (SD)	(62)	
Age	26.19(4.2)	24.55(3.6)	−1.691	0.096
NART (IQ)	43.42(5.3)	45.18(3.2)	1.532	0.13
Years of Education	19.20(3.14)	18.63(2.53)	−0.781	0.43
Gender	Number (%)	Number (%)	(1, 64)	0.99
Male	15(48)	16 (49)	?^2^(1, 64)	0.99
Female	16(52)	17(51)		

The table displays the demographic details for the participants in the study. Following randomization, the two treatment groups were not statistically significantly different in age (p = 0.096), years of education (p = 0.43), verbal intelligence (as evaluated with the NART; p = 0.13), or gender (p = 0.99).

The modafinil dose was well tolerated, with no adverse events reported. Compared to placebo, modafinil had no statistically significant effect on subjective mood on any of the measures of the VAS (p>0.1) (**Table 2**) or on pulse, or diastolic or systolic blood pressure (p>0.1) (**Table 3**).

**Table 2:** Mood Measures.

Visual Analogue Scales (VAS) Measure	Modafinil	Placebo	F Ratio^1^	P value	Partial Beta Squared (η*p* ^2^)
	Mean (SD)	Mean (SD)	(df = 1,40)		
Alert	69.44 (2.93)	69.08 (3.07)	0.007	0.9	0.00
Calm	65.92 (3.53)	60.57 (3.7)	1.091	0.3	0.27
Clear-headed	71.46 (2.74)	69.66 (2.8)	0.20	0.6	0.005
Well-Co-ordinated	71.39 (2.77)	69.51 (2.90)	0.22	0.6	0.005
Energetic	67.01 (2.95)	62.94 (3.10)	0.903	0.3	0.022
Contented	76.034 (2.93)	71.64 (3.07)	1.07	0.3	0.026
Tranquil	69.35 (3.06)	68.6 (3.21)	0.029	0.8	0.01
Quick-witted	67.27 (2.63)	67.35 (2.76)	0.00	0.9	0.00
Relaxed	66.78 (3.17)	64.78 (3.33)	0.18	0.6	0.005
Proficient	72.92 (2.42)	69.30 (2.54)	1.053	0.3	0.026
Happy	76.47 (2.99)	74.28 (3.14)	0.25	0.6	0.006
Amicable	77.76 (2.69)	75.30 (2.8)	0.39	0.5	0.010
Interested	76.08 (2.79)	72.58 (2.93)	0.74	0.3	0.018
Attentive	73.64 (2.841)	67.95 (2.91)	1.95	0.17	0.048
Strong	68.47 (3.04)	68.82 (3.19)	0.006	0.9	0.000
Gregarious	68.01 (2.896)	64.78 (3.03)	0.59	0.4	0.015

The table shows the effects of drug on self-reported mood. Compared to placebo, modafinil had no statistically significant effect on subject-reported mood on any of the measures of the VAS (p>0.1). ^1^Analysis of variance for repeated measures, df = (1,40).

**Table 3:** Cardiovascular Responses.

Cardiovascular Measures	Modafinil	Placebo	F Ratio^1^	P value	Partial Beta Squared (η*p* ^2^)
	Mean (SD)	Mean (SD)	(df = 4,180)		
Pulse	73(2.361)	68.6(2.10)	1.939	0.17	0.045
Systolic Blood Pressure	117.364(2.3)	121.800(2.17)	1.947	0.14	0.041
Diastolic Blood Pressure	67.54(1.61)	68.77(1.51)	0.31	0.581	0.007

The table shows the mean cardiovascular responses by the modafinil and the placebo group. Compared to placebo, modafinil had no statistically significant effect on pulse, diastolic or systolic blood pressure (p>0.1), or subject-reported mood on any of the measures of the VAS (p>0.1). ^1^Analysis of variance for repeated measures (df = 4,180).

### HSCT

As the HSCT requires quick and accurate verbal responses and activates brain areas that are dependent on language retrieval, semantic activation and selection in semantic search, three participants whose first language was not English were excluded from the HSCT data analysis.

### Latency

To test the hypothesis of whether modafinil increased the latency of verbal responses in the HSCT, a two-way mixed-model ANOVA using drug (placebo vs. modafinil) as a between-subject variable and the response latency of the test sections (Response Initiation; test section 1 vs. Response Inhibition; test section 2) as a within-subject variable (repeated measures) was conducted.

**Figure 2:**
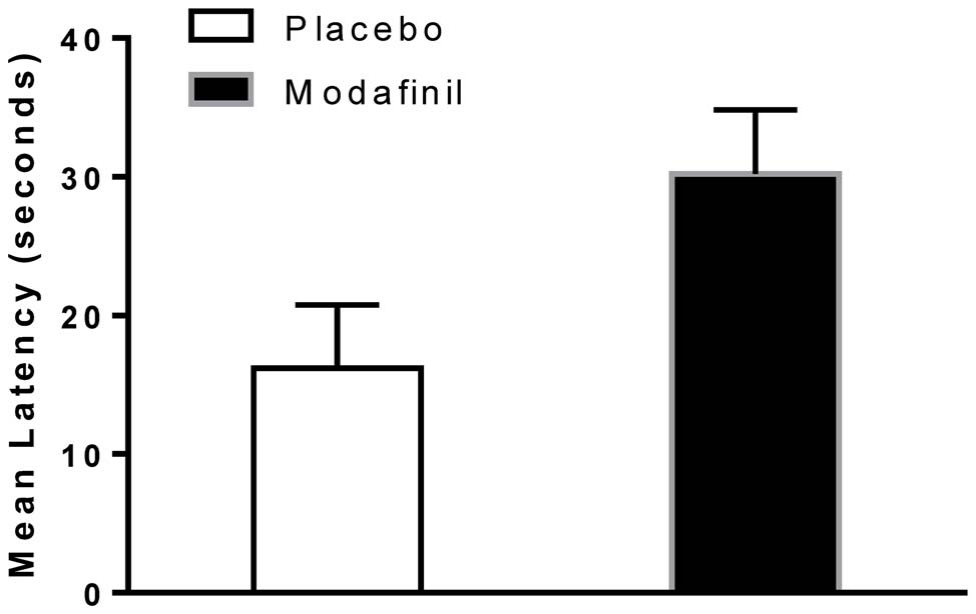
Latency. The figure depicts the effect of drug on the response latency in the performance of the Hayling Sentence Completion Task (HSCT). There was a significant effect of drug on latency in the completion of the task across sections, with modafinil-treated participants having longer response latencies across test sections (p = 0.035) **. Error bars represent the SEM.

This analysis revealed a significant main effect of test section (F(1,59) = 15.47, p<0.001, η*p* ^2^ = 0.20), with participants (across treatment groups) having longer latencies in the Response Inhibition section (Mean  = 32.92; SD = 36.98; 95% CI 23.45–42.39) compared to the Response Initiation section (Mean  = 13.30; SD = 27.09; 95% CI 6.36–20.23) of the task. There was a significant main effect of drug (F(1,59) = 4.674, p = 0.035, η*p* ^2^ = 0.73), with participants administered modafinil having longer response latencies across test sections (Mean  = 30.21; SD = 4.62; 95% CI 20.98–40.00) relative to placebo (Mean  = 16.22; SD = 4.54; 95% CI 7.14–25.30) (Figure 2). Because there was no statistically significant interaction between treatment group assignment and test sections for response latencies (F(1,59) = 0.022, p = 0.88, η*p* ^2^ = 0.0), no additional contrast analyses were conducted.

### Errors

**Figure 3:**
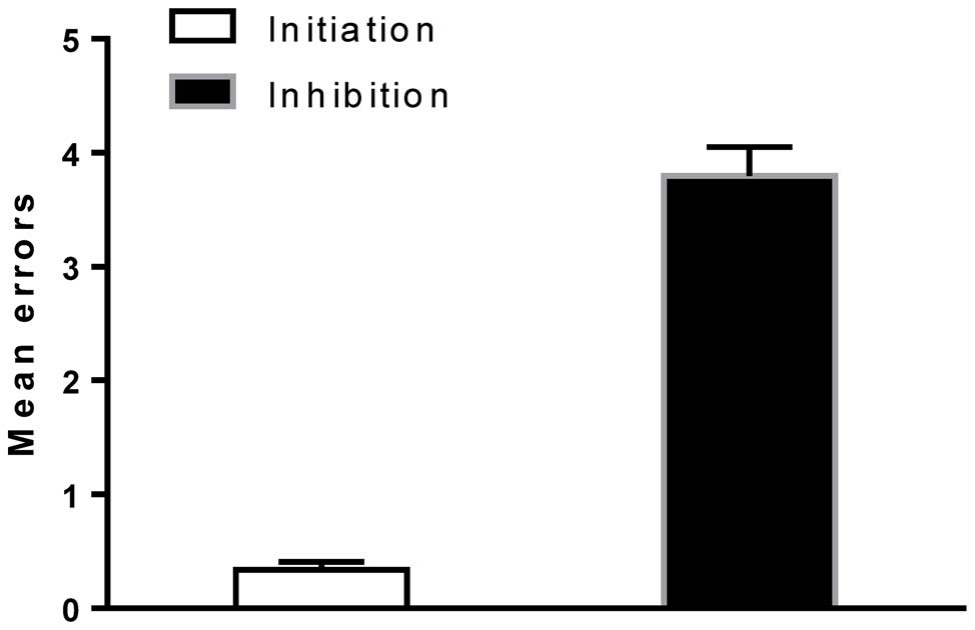
Errors. The figure depicts the effect of drug on errors in the performance of the Hayling Sentence Completion Task (HSCT). There was a significant effect of test section on errors committed during the task, with participants across treatment groups making more errors in the response inhibition section of the task relative to the response initiation section of the task. **P<0.01. Error bars represent the SEM.

To test the hypothesis of whether modafinil improved the accuracy of the performance of the HSCT, a two-way mixed-model ANOVA using drug (placebo vs. modafinil) as a between-subject variable and the errors committed in the test sections (Response Initiation; test section 1 vs. Response Inhibition; test section 2) as between-subject variable (repeated measures) was conducted. This analysis revealed a significant main effect of test section (F (1,59) = 167.541, p<0.001, η*p* ^2^ = 0.74), with participants across treatment groups making more errors in the Response Inhibition (Mean  = 3.8; SD = 1.9; 95% CI 3.30–4.30) section of the task relative to the Response Initiation section (Mean  = 0.34; SD = 0.6; 95% CI 0.19–0.50) (**Figure 3**). There was no significant main effect of drug on errors (F(1,59) = 0.34, p = 0.561, η*p* ^2^ = 0.006) or interaction between drug and errors committed in the test sections (F(1,59) = 0.22, p = 0.88, η*p* ^2^ = 0.023); therefore, additional contrast analyses were not conducted.

## Discussion

In this investigation of the effects of modafinil on the HSCT, a measure of executive function for context-appropriate language initiation and inhibition, we found that participants administered modafinil took significantly longer to perform the HSCT across task sections than placebo-treated participants, without showing any improvement with regard to errors on the task. Thus, in the present study, there was no evidence of delay-dependent cognitive enhancement by single 200 mg doses of modafinil.

The slowing effect of modafinil on the HSCT response latency measures is consistent with previous research indicating that modafinil slows latency of response during behavioural tasks in rats [21] and during the performance of cognitive decision-making tasks in patients diagnosed with ADHD [20], [54] and in healthy non-sleep deprived volunteers [18]. These observations suggest that some beneficial effects of modafinil (e.g., decreasing impulsive responses) may come at the cost of slowing responses in tasks that tap into frontal lobe function and require quick and accurate responses. The present findings indicate that such cognitive response slowing can occur without any commensurate benefit on other parameters of task performance, i.e., in reducing errors on the HSCT.

The lack of cognitive enhancing effects of modafinil on the HSCT is consistent with previous double-blind, placebo-controlled studies that investigated the effects of modafinil on several neuropsychological tasks but failed to find cognitive enhancing effects in healthy individuals [3], [15]–[18], [24], [55], [56]. Two recent reviews of the field also corroborate that modafinil does not always improve performance on several neuropsychological tasks [2], [25]. Furthermore, our findings are also consistent with a recent review [23] of several widely-used alleged cognitive enhancers, which suggested that, in studies with non-ADHD adults, psychostimulant medications do not promote acquisition of new information and might impair performance of tasks that require adaptation, flexibility and planning. The review concluded that the evidence does not support the conclusion that stimulants are cognitive ‘enhancers’ [23]. Recently findings on the effects of modafinil on creativity tasks in healthy volunteers support this review [24]. Taken together, this previous research raises the possibility that modafinil does not improve the performance of certain neuropsychological tasks because these tasks involve not only wakefulness and attentional components, but also sophisticated problem-solving abilities which modafinil may not be able to enhance [24], [29].

Alternatively, this published evidence may indicate that modafinil could have highly domain- specific actions, with some functions being enhanced while others are impaired and/or not affected [24]. In the current study, modafinil slowed the production of verbal responses on the HSCT without improving the accuracy of performance of the task. Of particular importance is the need to further clarify the robust observed slowing effect of modafinil in healthy individuals, because recent research has found that posterior inferolateral cortical injury also resulted, to a greater extent, in slowing of verbal responses in both the response initiation and inhibition sections of the HSCT [34]. Thus, it is still unclear how modafinil affects broad cognitive domains that are beyond attention and alertness. Hence, the mechanisms by which modafinil exerts its cognitive enhancing effects, and on what level it acts as a cognitive enhancing agent in healthy participants, are still unknown.

The goal of this study was to further explore the previously reported effect of modafinil in enhancing cognitive task performance while slowing performance of the task, which has been referred to as “delay-dependent cognitive enhancement”. This observation raised the question about whether modafinil was acting selectively as a cognitive enhancer, regardless of the temporal demands of the task, or whether the drug acts to slow responding less specifically, without necessarily providing any improvement in the quality of cognitive performance. The results of the present study suggest that the response-slowing effect of modafinil need not result in enhanced cognitive function, at least with respect to the frontal lobe-sensitive executive functions represented by accurate, time-efficient performance of the HSCT. This task is of particular interest with respect to modafinil because individuals with narcolepsy exhibit impaired performance on this task with respect to longer latencies to respond, as well as response errors [35]. In the present study, modafinil appeared not to enhance the accuracy of performance of the HSCT in healthy individuals but increased the latency of producing verbal responses to the task. These results argue against the notion that the slowing of response time in healthy volunteers is a necessary and sufficient condition for cognitive enhancement with modafinil.

An important objective of the present study was to explore the reported phenomenon of “delay-dependent cognitive enhancement” with modafinil, because the drug is reportedly widely used “off-label” by healthy individuals with the objective of improving cognitive performance (e.g., in school exams or in the work environment). Assuming that the perceived cognitive benefits of modafinil by off-label users are not merely a placebo response or secondary to an affective response, such as increased task enjoyment [57], [58], it is possible that these benefits are related to previously reported, possibly cognitive domain-specific effects of modafinil that are measured by certain neuropsychological tests (e.g., non-verbal response inhibition, measured with the stop-signal task [18]). Likewise, the response-slowing activity of modafinil, and the relationship of that effect to cognition, could also be domain-specific. In this context, to further understand the basis of modafinil users' reported impressions of enhanced cognition, it may be important to assess these potential benefits with cognitive tests that have demonstrated ecological validity [59] with respect to the school or work environment in which users have reportedly experienced benefits [13]. Although we cannot exclude the possibility that such enhancement could have been seen at a lower dose (e.g., 100 mg) or higher doses than employed in the present study, previous studies demonstrating both delays in responding and cognitive enhancement on selected tasks have reported these effects in a dose-independent manner at both 100 and 200 mg doses of modafinil in healthy participants [18].

Finally, since slowed responding in the performance of the HSTC due to modafinil administration is the key finding of this study, it is appropriate to consider the accuracy of the time measurement method used in the study. Previous investigations of the reliability and accuracy of handheld stopwatches compared to electronic timing methods have indicated very high intra-class correlations between these methods, approaching 0.99 [60], [61]. In addition, the response times were captured by the experimenter with a stopwatch with 1/100 second lap and split timing accuracy, and the mean response times of the modafinil group were almost double the mean response times of the placebo group, which are much greater than the demonstrated measurement error for stopwatches. Hence, it is unlikely that the findings were influenced by how the experimenter captured the data. The findings suggest that that modafinil increases the latency of response in the HSCT in healthy volunteers, a phenomenon which requires additional study.

## Conclusions

In conclusion, the goal of this study was to investigate the effects of modafinil on the HSCT, which is sensitive to frontal lobe function. Our results show that relative to placebo, participants administered modafinil were significantly slower in the performance of the task overall. This finding is consistent with previous research indicating that modafinil slows latency of response during cognitive tasks in patients diagnosed with ADHD and in healthy non-sleep deprived volunteers. The lack of improvement in accuracy of performance on the HSCT following modafinil administration extends previous research with healthy volunteers that also failed to find cognitive-enhancing effects of modafinil on several neuropsychological tasks. We suggest that future studies should investigate the effects of modafinil with testing procedures that involve not only basic executive functions but also cognitive flexibility, language retrieval, and creativity, as well as tasks that exhibit “ecological validity” with respect to the demanding environments in which this drug is often used. Investigating these higher order cognitive processes is likely to lead to better understanding of the effects of modafinil on cognition in healthy individuals. In sum, the current study shows that modafinil increases the latency of response to the HSCT without improving accuracy of the task, and thus provides new information to better define the limits of cognitive enhancement with this drug.

## Supporting Information

Checklist S1 **CONSORT Checklist.**
(PDF)

Protocol S1 **Trial Protocol.**
(DOC)
