# Supplementary material for: Modafinil Increases the Latency of Response in the Hayling Sentence Completion Test in Healthy Volunteers: A Randomised Controlled Trial
Source: PLoS One. 2014 Nov 12;9(11):e110639. doi: 10.1371/journal.pone.0110639 (PMC4229110; doi:10.1371/journal.pone.0110639)
Supplement: Protocol S1 — Trial Protocol. (DOC) [file pone.0110639.s002.doc]

Research Protocol

THE EFFECT OF MODAFINIL ON CONGITION AND SUBJECTIVE WELLBEING IN HEALTHY ADULTS

Chief and Principal Investigator
Professor Barbara Sahakian,
Professor of Clinical Neuropsychiatry,
Cambridge School of Clinical Medicine &
Behavioural and Clinical Neuroscience Institute
University of Cambridge,
Department of Psychiatry,
Addenbrooke's Hospital
Hills Road, Cambridge
CB2 2QQ
Tel: +44(0)1223 331209
Email: ma114@hermes.cam.ac.uk

Research Team:

Professor Trevor Robbins, Department of Experimental Psychology & BCNI, University of Cambridge (Head of Experimental Psychology, University of Cambridge)

Dr James Rowe, Department of Clinical Neurosciences & BCNI, Honorary Consultant neurologist at the Cambridge University Neurology Unit & MRC Cognition and Brain Sciences Unit

Mr Ahmed D. Mohamed, Department of Psychiatry & BCNI, University of Cambridge,

Aims of the Project

This project aims to investigate the effect of modafinil on cognitive performance, divergent thinking and subjective well-being in healthy participants. The main task for this project is to address whether the same effects of acutely administered modafinil on cognitive function are observed across four testing sessions or whether its effects exhibit tolerance. Participants' divergent thinking, cognitive performance and subjective well-being will be investigated in a between-subjects parallel design study.

Two groups each of 32 healthy participants will attend four separate sessions at the Wellcome Trust Clinical Research Facility (WTCRF), during which they will be tested on objective measures of divergent thinking tasks, a computerised neuropsychological battery, and on well-validated questionnaires quantifying subjective well-being. Participants will be randomly allocated to one group and will consistently receive either a dose of modafinil (200mg) (Modafinil group) or a placebo (Control group) prior to testing on all four sessions.

All subjects will undertake CANTAB tests and different versions of the divergent thinking tasks. Based on the evidence that modafinil enhances executive planning and working memory in healthy adults (Turner et al 2003), we predict that healthy individuals in the Modafinil group will perform better in the divergent thinking tasks and in the cognitive performance tasks. Furthermore, we expect a specific subjective well-being effect to be associated with recurrent modafinil use in healthy young adults.

Background

Modafinil (Provigil, 1997) is a novel drug which has a demonstrable efficacy in the treatment of daytime sleepiness associated with narcolepsy (Benerjee et al., 2004). Studies with healthy volunteers,

including those conducted by our research group, showed that modafinil improves some aspects of neuropsychological task performance, possibly through improved inhibitory control (Turner et al., 2003; Baranski et al., 2004; Muller et al., 2004; Randall et al., 2005). For instance, Turner et al. (2003) assessed the cognitive enhancement potential of modafinil on sixty young healthy male volunteers who either received a single oral dose of placebo, or 100mg, or 200mg of modafinil before undertaking tasks on working memory and attention. Modafinil significantly improved performance of digit span, visual pattern recognition memory, spatial planning and stop signal reaction time. It also significantly improved performance on tests on decision making and delayed matching to sample.

Modafinil also had a significant beneficial effect on working memory and executive attention in patients with neuropsychiatric disorders including depression (Padala, Burke, & Bhatia, 2007), schizophrenia (Turner et al., 2004b), and Parkinson's disease (Happe, Pirker, Sauter, Klosch, & Zeitlhofer, 2001). Turner et al., (2004a) also found that modafinil enhanced cognitive functioning in ADHD patients, who after 2-3 hour post administration of modafinil, significantly improved on tests of short-term memory span, visual memory, spatial planning, and stop-signal motor inhibition. On several measures, modafinil increased sustained attention accuracy whilst slowing response latency.

However, most previous studies have significant shortcomings, as they only investigated the acute effect of modafinil on cognition and therefore are not able infer whether the same effects modafinil on cognitive function are consistently observed in subsequent testing sessions (Pubmed review 07/12/2009).

Our aims are (i) to further characterise possible cognitive enhancing effects of modafinil by using a broader range of test (including divergent thinking, or 'creativity') and a more difficult cognitive test battery to avoid possible ceiling effects on some tests and determine whether the beneficial effects of modafinil are in a restricted range of tests or functions as appears previously to have been the case. (ii) to test whether any drug effects exhibit behavioural tolerance, i.e. diminishing with repeated testing. These aims will be achieved with a double blind, placebo controlled study that measures participants' cognitive performance over four weekly sessions.

We will investigate the effect either of one weekly dose of modafinil (200mg) or placebo for four sessions on tests of divergent thinking, cognitive performance and subjective well-being in healthy adults. This will be accomplished by employing reliable divergent thinking tasks, a well-validated cognitive battery and a sensitive subjective well-being questionnaire.

Predictions with regard to the research questions/objectives:

1. **For Cognitive and Divergent thinking**

- We predict that participants treated with modafinil will perform significantly better in the above named cognitive tasks and divergent thinking compared with the placebo group.
- The effect of modafinil on divergent thinking is difficult to predict but it is expected that there will be an improvement at least in the first session.
- Modafinil is efficacious for sustaining and restoring objective performance and alertness in sleep deprived and non sleep deprived humans (Wesensten, 2006), it is therefore predicted that modafinil will have a significant effect (two-tailed) on ratings of subjective well-being in healthy adults.
- We expect that modafinil will have a greater significant positive effect on subjective well-being and divergent thinking in healthy participants with low scores at baseline compared to healthy participants with high scores at baseline.

2. **First session compared with the following session**

- Finally, it is predict that there will be a significant difference between the effect of modafinil on divergent thinking scores, cognitive performance and subjective well-being in the first testing session in comparison to the other three sessions.

<u>Methods:</u>

Study Design: This is a double-blind, placebo-controlled, between subjects, parallel design study. 2 x 30 participants will receive either a single oral dose of a lactose placebo or 200mg of modafinil for four occasions (one dose per week to allow for drug washout). Participants will either be identified via the Behavioural and Clinical Neuroscience Institute participant panel or via an advertisement. Once potential participants are interested in taking part in the study, they will be sent an information sheet and contacted within 24 hours. Participants will be informed of the details of the study and will have the opportunity to ask questions or withdraw from it at any time.

Exclusion criteria: Prior to the first testing session, possible participants will be screened through telephone and any participant with any significant psychiatric history, visual or motor impairment or the concurrent use of any psychotropic medications or any medication contra-indicated with modafinil will be excluded. In addition, participants with a history of hypertension, cardiac disorders, epilepsy or drug or alcohol abuse will also be excluded. The two groups will be matched for age, verbal abilities by using the National Adult Reading Test, (Nelson, 1991) and education level. All participants will be advised not to consume alcohol or caffeine-containing drinks for 12 hours before the study.

Testing procedure: On a convenient date, participants will be asked to attend the WTCRF at Addenbrooke's Hospital on four occasions, separated by a maximum of one week. Once participants arrive at the WTCRF at about 8:30am, we will confirm their details and ask them to complete a personality and NART measures. After that, we will measure their blood pressure. Then we will give participants some capsules to swallow with water. These capsules will contain either a drug (modafinil), or a placebo (an inert lactose pill) –neither the participant nor the researcher testing the participant will know which pill participants are given on a particular day. A physician (Dr J Rowe) will be available to provide medical assessment if participants experience any adverse events related to participation.

After ingesting the pill, participants will then rest in a quiet area where they can read books or magazines, and watch television for one and a half hours. Similar to previous research, participants will be tested 2 hours post-drug administration, for approximately 2.5 hours. At 2h participants will complete a divergent thinking task, a computerized cognitive performance task, and two subjective well being questionnaires. During the computerized testing, we will measure their blood pressure again.

After the first session, participants will return to the research facility for three more times separated by a week. In the subsequent three sessions, participants will undertake further sessions which will be similar in procedure but using different versions of the tests where possible. A psychologist will instruct the volunteers on how to perform each task and will provide feedback if necessary.

Measures **Total Testing time (120 min)**

Measures of the divergent thinking will be drawn from the following:

- Match Stick problem (Knoblich et al., 1999) which measures how healthy adults learn to override the imperatives of past experience in the face of novel conditions (12 min).

- Wallach and Kogan's (1965) test of divergent thinking task assessing pattern meaning, line drawings, similarities and alternative uses tasks (10 min).
- A short version of the original Hayling task (Burgess and Shallice, 1997) that measures cognitive flexibility (7 min).

Cognitive measures will be drawn from the well established CANTAB neuropsychological test battery: (Sahakian and Owen, 1992-www.camcog.com). All participants will receive the same tests in the same order. All computerised tasks will be run on an Advantech personal computer (Model PPC-120T-RT), and responses will be registered either via the touch-sensitive screen or a response key, depending on the task. The following cognitive test battery will consist of:

The Stop Signal Test (Logan, Cowan et al. 1984), a 20 minute task assessing the inhibition of motor responses to an auditory 'Stop' signal.

- Reward sensitivity (Cools et al. 2005), a 15 minute reaction time task assessing ability to respond to changing reward value.
- A version of 5-Choice Serial Reaction Task which is suitable for humans (Robbins, 2002) (5 min).
- Digit Span assessing working memory and vigilance.(5 min)
- The Paired Associates Learning (PAL) task which tests the ability to learn and form visuo-spatial associations (Sahakian et al., 1988) (10 min)
- Pattern Recognition Memory (PRM) which is a two choice test of abstract visual pattern recognition memory (Mehta et al., 1999) (5 min).
- The Delayed Matching to Sample (DMTS) which is a 4 choice of simultaneous and delayed matching to sample abstract pattern task with distractors (Robbin et al., 1994) (10 min).
- The Rapid Visual Information Processing (RVIP) task measuring sustained attention (7 min)
- The 'one-touch' Tower of London spatial planning task (NTOL) which measures planning (Owen et al., 1995) (10 min) difficult version

Short self-report questionnaires, consisting of

- SF-8: SF-8 to measure participants' subjective wellbeing (5 min).
- Creative Personality Scale (CPS) for the Adjective Checklist by Gough's (1979) measuring whether participants are high or low in creativity(7 min).
- Visual Analogue Scale (VAS) (Bond & Lader, 1974) which assesses participants mood before, during and after the tablet administration (2 min).

Sample Size and Analysis: The Sample 30 per group was estimated to provide sufficient statistical power based on previous within-subject medication studies (Turner, 2003;). For a large effect size of modafinil study (d=0.52), the statistical power software (GPower) indicates a power of 0.95 to detect a large effect size with 0.05 error (i.e type I false positive error 5%) with 30 participants. A stronger interaction between modafinil and group (modafinil vs placebo x group) with a large effect size d=0.52, for false positives (a=0.05) with 30 participants in each group yields a power of 0.95. An interim analysis will be performed after the completion of the first sixteen participants in each group

Statistical analysis: All data will be analysed using Windows versions of SPSS (Version 15). To investigate the effect of experimental treatment on test performance, differences between group mean (or median) performance for group scores will be analysed using a one-way and repeated analysis of variance (ANOVA) or the equivalent non-parametric Kruskal-Wallis ANOVA. To clarify the nature of any such differences, planned orthogonal contrasts comparing the effect of modafinil, and the effect of placebo with the drug group, will be performed where appropriate. In instances where

several readings are taken for the same score, repeated measures ANOVA will be used to test the effects of relevant independent within- and between-subjects variables. As our hypotheses are both one and two directional, all tests will employ two-tailed statistics threshold at $p<0.05$.

This is small scale study using modafinil as a pharmacological tool to neuromodulate cognitive performance and subjective wellbeing in healthy adults .It is therefore not a clinical trial under MHRA definitions.

***Main Ethical Considerations*:**

Several areas have been considered here.

1. Confidentiality. We follow standard good research practices within our departments, to ensure confidentiality of electronic and hard copy data, in keeping with the Data Protection Act. Hard data are kept locked, and electronic data are anonymised and encrypted. We collaborate with other members of the University and require similar adherence to DPA and confidentiality by collaborators. We do not anticipate data sharing outside the UK. Data will be stored for maximum of 10 years within the department.
2. Safety. The safety of participants is paramount. This study involves a single standard dose (200mg) of modafinil, a medication with an excellent safety profile that is currently licensed for the treatment of daytime sleepiness. Modafinil has been used previously in multiple LREC−approved studies including male and female participants within the Department of Psychiatry (University of Cambridge) with no adverse reactions or problems reported. We have used modafinil with both patients (Backwell et al., 2008, CamREC reference number 04/076; Turner et al., 2004a, b) and Healthy adults (Winder-Rhodes et al., 2009-CamREC reference number 06/Q0106/47; Turner et al., 2003) and consistent with research evidence modafinil is safe (Deroche-Gamonet et al 2002; Billiard et al 1994) and current dose of modafinil has been well tolerated in studies run by members of our research team (e.g., Turner et al., 2009; Turner et al., 2004b). Furthermore, patients who are taking other forms of psychiatric or non−psychiatric medications contraindicated for use with modafinil will not be included in this study. The structured health interview as well as the information provided to participants will screen for pregnancy, breast−feeding, and volunteers who are attempting to become pregnant (see protocol and information sheet).

   Participants will be screened via questionnaire to ensure they have no history of relevant medical problems (e.g. hypertension, heart disease), and are not taking other medications which might adversely interact with modafinil. A physician (Dr J Rowe) will be available if participants experience any adverse events related to participation.

3. Consent. All participants will, by definition, have the capacity to provide informed consent. As this study investigates healthy young participants, vulnerable participants, including pregnant women, will not be included in this study. Consent procedures include enough information and enough time to make a decision to participate, and we stress the voluntary nature of participation and ability to withdraw. We have written our information sheets and consent sheets so as to be complete, but concise and

comprehensive for the lay participant. In addition, participants will have the ultimate decision to participate in this study.

4. Comfort. Furthermore, there is no reason to believe that the neuropsychological testing procedure should carry any adverse consequences for the health of participants. Each task takes under 20 minutes; all tasks are administered by a trained researcher with psychology experience to at least post-graduate level and clinical experience.
   The cognitive testing battery requires participants to interact with a computer equipped with a touch-sensitive screen. Participants will perform the cognitive testing battery for approximately 2.5 hours. Short breaks will be included throughout the battery, and participants will be reminded that they can take a break at any time by asking the researcher. The procedures used in this experiment will neither be physically stressful nor impinge on the safety of the participants. The images and feedback that are presented are also not emotional and have not caused any distress in related studies of patients or healthy controls. Testing will stop if a participant reports excessive frustration or appears tired.
5. Withdrawal. All participants are invited to participate in 2.5 hours testing time in each session. Participants will be paid £25 for each session. However, it will be made clear to all participants that they may withdraw during the session at any time without any consequences for their future care. All necessary arrangements will be made to ensure that participants are at all times comfortable. It will be made clear to all participants that while they do not stand to directly benefit from the experimental procedure, the results of the study will contribute to our understanding of the efficacy of drugs for healthy adults and patients with neuropsychiatric disorders.
6.

References

Arnsten, A. and T. W. Robbins (2000). Neurocehmical modulation of prefrontal function in humans and animals. Principles of Frontal lobe function. S. DT and K. RT. New York, Oxford University Press: 51-84.

Chemelli RM, Willie JT, Sinton CM, Elmquist JK, Scammell T, Lee C, et al. (1999). Narcolepsy in orexin knockout mice: molecular genetics of sleep regulation. Cell, 98(4), 437-451.

de Saint Hilaire, Z., Orosco, M., Rouch, C., Blanc, G., & Nicolaidis, S. (2001). Variations in extracellular monoamines in the prefrontal cortex and medial hypothalamus after modafinil administration: a microdialysis study in rats. Neuroreport, 12, 3533-3537.

de Saint Hilarie, Z., Orosco, M., Rouch, C., Blanc, G., & Nicolaidis, S. (2001). Variation in extracellular monoamines in the prefrontal cortex and medial hypothalamus after modafinil administration: A Microdialysis Study in Rats. Neuroreport, 12(3533-3537).

Farah, M., Caroline, H., Sankoorikal, G., Smith, M. E., & Chatterjee, A. (2009). When we can enhance cognition with Adderall, do we sacrifice creativity? Psychopharmacology, 203(3), 541-547.

Goldberg, D. P., & Williams, P. (1988). The User's Guide to the General Health Questionnaire. Windsor: NFER-Nelson.

Happe, S., Pirker, W., Sauter, C., Klosch, G., & Zeitlhofer, F. (2001). Successful treatment of excessive daytime sleepiness in parkinson's disease with modafinil. Journal of Neurology, 248, 632-634.

Hieble, J. P., Bondinell, W. E., & Ruffolo Jr, R. R. (1995). Alpha- and beta-adrenoceptors: from the gene to the clinic. 1. Molecular biology and adrenoceptor subclassification. J Med Chem, 38, 3415-3444.

Hu, Y., Stewart-Brown, S., Twigg, L., & Weich, S. (2007). Can the 12 Item General Health Questionnaire be used to measure positive mental health? Psychological Medicine, 37(7), 1005-1013.
Huppert, F. A., & Whittington, J. E. (2003). Evidence for the independence of postive and negative well-being: Implications for quality of life assessment. British Journal of Health Psychology 8, 107-122.
Jasinski, D. (2000). An Evaluation of the Abuse of Modafinil using methylphenidate as a reference. Journal of Psychopharmacology, 14, 53.
Lanni, C., Lenzken, S. C., Pascale, A., Del Vecchio, I., Racchi, M., Pistoia, F., et al. (2008). Cognition enhancers between treating and doping the mind. Psychopharmacologial Research, 57, 196-213.
Logan, G. (1994). The ability to inhibit thought and action. . In Dagenbach, D, Carr, TH, Inhibitory Process in attention, memory and language. (pp. 198-239). San Diego: Academic Press.
Madras, B., Xie, Z., Jassen, A., Panas, H., Lynch, L., Johnson, R., et al. (2006). Modafinil occupies dopamine and norepinephrine transporters in vivo and modulates the transporter and trace amine activity in vitro. J Pharmacol Exp Ther., 319, 561-569.
Minzenberg, M. J., & Carter, C. S. (2008). Modafinil: A review of Neurochemical Action and Effects on Cognition. Neuropsychopharmacology, 33, 1477-1502.
Minzenberg, M. J., & Cater, C. S. (2007). Modafinil: A review of Neurochemical Actions and Effects on Cognition. Neuropsychopharmacology, 1-26.
Muller, U., Steffenhagen, N., Regenthal, R., & Bublak, P. (2004). Effects of modafinil on working memory processes in humans. Psychopharmacology, 177, 161-169.
Owen, A., Sahakian, B. J., Semple, J., Polkey, C., & Robbins, T. W. (1995). Visuo-Spatial short-term recognition memory and learning after temporal lobe excicion, frontal love excisions or amygdalo-hippocapectomy in man. . Neuropsychologia, 13, 1-34.
Padala, P. R., Burke, W. J., & Bhatia, S. C. (2007). Modafinil Therapy for Apathy in an Elderly Patient. The annals of Pharmacology, 41(2), 346-349.
Prabhakaran, V., smith, A. L. J., Desmond, J. E., Glover, G. H., & Gabrieli, J. D. E. (1997). Neural Substrates of Fluid Reasoning: An fMRI Study of Neocortical Activation during Performance of the Raven's Progressive Matrices Test. Cognitive Psychology, 33, 43-63.
Provigil. (1997). Modafinil Data Sheet. Data for the First Authorisation.
Raven, J. C. (1976). Standard Progressive Matrices: Sets A, B, C, D, & E. Oxford: Oxford University Press.
Robbins, T. W. (2009). Special Issue on Cognitive Enhancers. Psychopharmacology, 202, 1-2.
Robbins, T.W.,(2000). From arousal to cognition: the integrative position of the prefrontal cortex. Prog Brain Res,. 126: p.469−83.
Rogers, R., Blackshaw, A., Middleton, H., Matthews, K., Hawtin, K., Crowley, C., et al. (1999). Tryptophan impaires stimulus reward learning while methylphenidate disrupts attentional reward learning in healthy young adults: Implication for the monoaminergic basis of impulsive behaviour. Psychopharmacology, 146, 486-491.
Rosenthal, M. H., & Braant, S. (2003). Benefits of adjunct Modafinil in an open label, pilot study in patients with Schizophrenia. Schizophrenia Research, 60(1), 301.
Rubin, R. (2009). A warning on off-label use of sleep-disorder drug (pp. 1).
Sahakian, B. J., & Morein-Zamir, S. (2007). Professor's Little Helper. Nature, 450(20), 1157-1159.
Turner, D. C., Clark, L., Dawson, J., Robbins, T. W., & Sahakian, B. J. (2004a). Modafinil Improves cognition and response inhibition in adult attention-deficit Hyperactive disorder. Biological Psychiatry, 55, 1031-1040

Turner, D. C., Clark, L., Pomarol-Clotet, E., McKenna, P., Robbins, T. W., & Sahakian, B. J. (2004b). Modafinil improves cognition and attention set shifting in patients with Chronic Schizophrenia. Neuropharmacology, 29, 1363-1373.
Turner, D. C., Robbins, T. W., Clark, L., Aron, A. R., Dowson, J., & Sahakian, B. J. (2003). Cognitive enhancing effects of modafinil in healthy volunteers. Psychopharmacology, 165, 260-269.
Walsh, J. K., Randazzo, A. C., Stone, K. L., & Schweitzer, P. K. (2004). Modafinil improves alertness, vigilance, and executive function during simulated night shifts. Sleep, 27, 434-439.
Wesensten, N. J. (2006). Effects of modafinil on cognitive performance and alertness during sleep deprivation. Curr Pharm Des, 12, 2457-2471.
Winder-Rhodes et al?
